# Co-infections with *Babesia microti* and *Plasmodium* parasites along the China-Myanmar border

**DOI:** 10.1186/2049-9957-2-24

**Published:** 2013-10-03

**Authors:** Xia Zhou, Sheng-Guo Li, Shen-Bo Chen, Jia-Zhi Wang, Bin Xu, He-Jun Zhou, Hong-Xiang Zhu Ge, Jun-Hu Chen, Wei Hu

**Affiliations:** 1 National Institute of Parasitic Diseases, Chinese Center for Disease Control and Prevention, WHO Collaborating Centre for Malaria, Schistosomiasis and Filariasis, Key Laboratory of Parasite & Vector Biology Ministry of Health, Shanghai 200025, China; 2 Department of Parasitology, Medical College of Soochow University, No.199 Renai Road, Suzhou 215123, China; 3 Tengchong Center for Disease Control and prevention, Yunnan province 679100, China; 4 Department of Microbiology and Microbial Engineering, School of Life Science, Fudan University, Shanghai 200433, China

**Keywords:** *Babesia*, *Plasmodium*, Co-infection, China-Myanmar border

## Abstract

**Background:**

Babesiosis is an emerging health risk in several parts of the world. However, little is known about the prevalence of *Babesia* in malaria-endemic countries. The area along the China-Myanmar border in Yunnan is a main endemic area of malaria in P.R. China, however, human infection with *Babesia microti* (*B. microti*) is not recognized in this region, and its profile of co-infection is not yet clear.

**Methods:**

To understand its profile of co-infections with *B. microti*, our investigation was undertaken in the malaria-endemic area along the China-Myanmar border in Yunnan between April 2012 and June 2013. Four parasite species, including *B. microti, Plasmodium falciparum* (*P. falciparum*), *P. vivax,* and *P. malariae,* were identified among 449 suspected febrile persons detected by nested polymerase chain reaction (PCR) assay based on small subunit ribosomal ribonucleic acid (RNA) genes of *B. microti* and *Plasmodium* spp.

**Results:**

Of all the collected samples from febrile patients, mono-infection with *B. microti, P. vivax*, *P. falciparum*, and *P. malariae* accounted for 1.8% (8/449), 9.8% (44/449), 2.9% (13/449), and 0.2% (1/449), respectively. The rate of mixed infections of *B. microti* with *P. falciparum* or *P. vivax* are both 0.2% (1/449), and mixed infections of *P. falciparum* and *P. vivax* accounted for 1.1% (5/449).

**Conclusions:**

This report supports the hypothesis that babesiosis caused by *B. microti* is emerging along the China-Myanmar border in the Yunnan province, P.R. China, but it was ignored because of low parasitemia or mixed infection with *Plasmodium* spp. More sensitive and specific diagnosis methods are needed to find the rapid response mechanism of emergency for babesiosis and malaria co-prevalence areas.

## Multilingual abstracts

Please see Additional file 1 for translations of the abstract into the six official working languages of the United Nations.

## Background

Babesiosis is a typical zoonotic disease which is caused by *Babesia* spp*.*, tick-borne intraerythrocytic protozoan parasites. The first definitive case of human babesiosis, was documented in a Yugoslavian farmer in 1957 [1]. The first case in an immunocompetent person was identified on Nantucket Island, off the coast of Massachusetts, in 1969 [2]. In fact, the causative agent was *Babesia microti*, and the vector was the *Ixodes dammini* tick (now referred to as *I. scapularis*) [3]. Babesiosis is now classified as a notifiable disease and is recognized as an emerging health risk in several parts of the world [4], but little is known about the its prevalence in malaria-endemic countries, where misidentification as *Plasmodium* spp. probably  occurs  (http://dpd.cdc.gov/dpdx/HTML/Babesiosis.htm). In Asia, *B. microti*-like organisms have caused illness in Japan, Taiwan, and mainland China [5-8]. In Europe, a few human babesiosis cases have been attributed to *B. divergens*, the cattle species transmitted by *I. ricinus*[9-11]. Hundreds of human babesiosis cases in the northeast and midwest United States have been attributed to *B. microti*[12]*.* Sporadic cases of babesiosis have been reported in Africa, Australia, and South America [9,13,14].

Recently, co-infection of parasites in humans has been noticed in many areas [15-17]. Human babesiosis has sometimes been diagnosed initially as malaria because of the similarity between the two diseases or the two parasites [18,19]. Thus, it is likely that cases of human babesiosis in countries in which malaria is endemic have been overlooked or misdiagnosed as malaria. The area along the China-Myanmar border in Yunnan has been reported as a main endemic area of malaria in the People’s Republic of China (P.R. China) [20,21], however *B. microti* has not been recognized as being endemic in this region in the past. Therefore, surveillance was carried out between April 2012 and June 2013 on the presence of *B. microti* from the patients’ blood as detected by molecular tools collected in this area.

## Methods

### Study area and population

The area along the China-Myanmar border in Yunnan is a main endemic area of malaria in China. The region is hilly and covered largely by primary and secondary rainforests. Inhabitants of the division are mainly rural indigenous people. Besides working for the logging industry in the surrounding jungles, inhabitants of the Tengchong county in the Yunnan province are actively involved in mining, farming, hunting, logging, and working abroad in Myanmar but coming back frequently. The population of the Tengchong county is served by a general hospital, a polyclinic at the county’s Center for Disease Control and Prevention (CDC), and 15 government health clinics.

The study was reviewed and approved by the Ethics Committee at the National Institute of Parasitic Diseases, Chinese Center for Disease Control and Prevention. Four hundred and forty-nine blood samples in people with malaria-like symptoms were obtained between April 2012 and June 2013 in the area along the China-Myanmar border in the Yunnan province of China. Written consent was obtained from each person before blood samples were obtained.

### Blood samples

This survey was carried out on the population seeking care at the Tengchong county CDC. During the study period, finger-prick blood samples on filter paper from 449 febrile patients with malaria-like symptoms were taken. The thick and thin blood films were stained with Giemsa and examined by staff at the diagnostic laboratories of the polyclinic and hospital.

A drop (20 μL-50 μL) of blood was placed directly on a pre-marked filter paper. DNA was extracted from the blood spots with QIAGEN (QIAamp DNA Blood Mini Kit, Biosearch Technologies, Inc. USA) and all of the positive samples were used for sequencing the small subunit ribosomal RNA (SSU rRNA) of the parasites.

### SSU rRNA Sequence and phylogenetic analysis

After the screening of *Plasmodium* spp. infection by microscopy, all the samples’ DNA templates for a nested polymerase chain reaction (PCR) were prepared from whole blood spots on the filter paper. Two microliters of PCR product from each, amplified by *Plasmodium* genus-specific primer pair rPLU1 and rPLU5, were subjected to a second PCR amplification with species-specific primer pairs rVIV1 and rVIV2 for *P. vivax*, rFAL1 and rFAL2 for *P. falciparum*, rMAL1 and rMAL2 for *P. malariae*, and rOVA1 and rOVA2 for *P. ovale*[22]. *B. microti*-specific fragments of SSU rRNA were amplified from the DNA extracted from all the febrile patients by nested PCR with two sets of *B. microti*-specific primers, Bab1-Bab4, and Bab2-Bab3 (*B. microti* detection PCR), in accordance with the method established by Persing *et al.*[23]. For *B. microti* detection PCR-positive samples, a more accurate analysis by amplification of the full-sized sequence (~1.7 kb) encoding the 18S rRNA gene of the *Babesia* parasite from the patients was applied with the nested PCR primers to confirm the infections. The first round primers were Piro1F: 5’-CCATGCATGTCTWAGTAYAARCTTTTA-3’ and rRNA-3’: 5’-ATCCTTCYGCAGGTTCACCTAC-3’. The second round primers were BablA: 5’-GTCTTAGTATAAGCTTTTATACAGCG-3’ and Prio6R: 5’-CTCCTTCCTY TAAGTGATAAGGTTCAC-3’, as described by Medlin *et al.*[24,25]. PCR amplification for each sample was done in a 50 μL reaction mixture containing 400 mM each of deoxynucleoside triphosphates, 0.25 mM of primer, ten to 100 ng of template DNA, and 2.5 U of Taq DNA polymerase (TIANGEN Biochemical) in 50 ml of the PCR buffer supplied together with the enzyme. PCR amplification parameters were: 94°C for four minutes, 35 cycles at 94°C for 30 seconds, 55°C for one minute and 72°C for 2 min, followed by a final extension for ten minutes at 72°C. PCR products amplified with nested primers were analyzed by agarose gelelectrophoresis. DNA bands were removed from the gel, purified by using the QIAquick Gel Extraction Kit (QIAGEN, Valencia, CA, USA), and ligated to T-cloning vector (Invitrogen, Carlsbad, CA, USA) according to protocols provided by the manufacturers.

To avoid errors caused by contamination of PCR products, DNA was extracted and divided into several aliquots in a safety cabinet placed in a room in which PCR products had never been treated. Detection and confirmation of PCR assays were separately carried out with the different aliquots.

Plasmid inserts were then sequenced. Sequence identity was confirmed by random basic local alignment search tool analysis of sequences in GenBank (http://blast.ncbi.nlm.nih.gov/). Phylogenetic relationships of unique sequences amplified were constructed by using the neighbor-joining method using MegAlign software (DNASTAR, Inc. Madison, WI, USA).

## Results

### Detection of B. *microti* and *Plasmodium* spp

**Figure 1:**
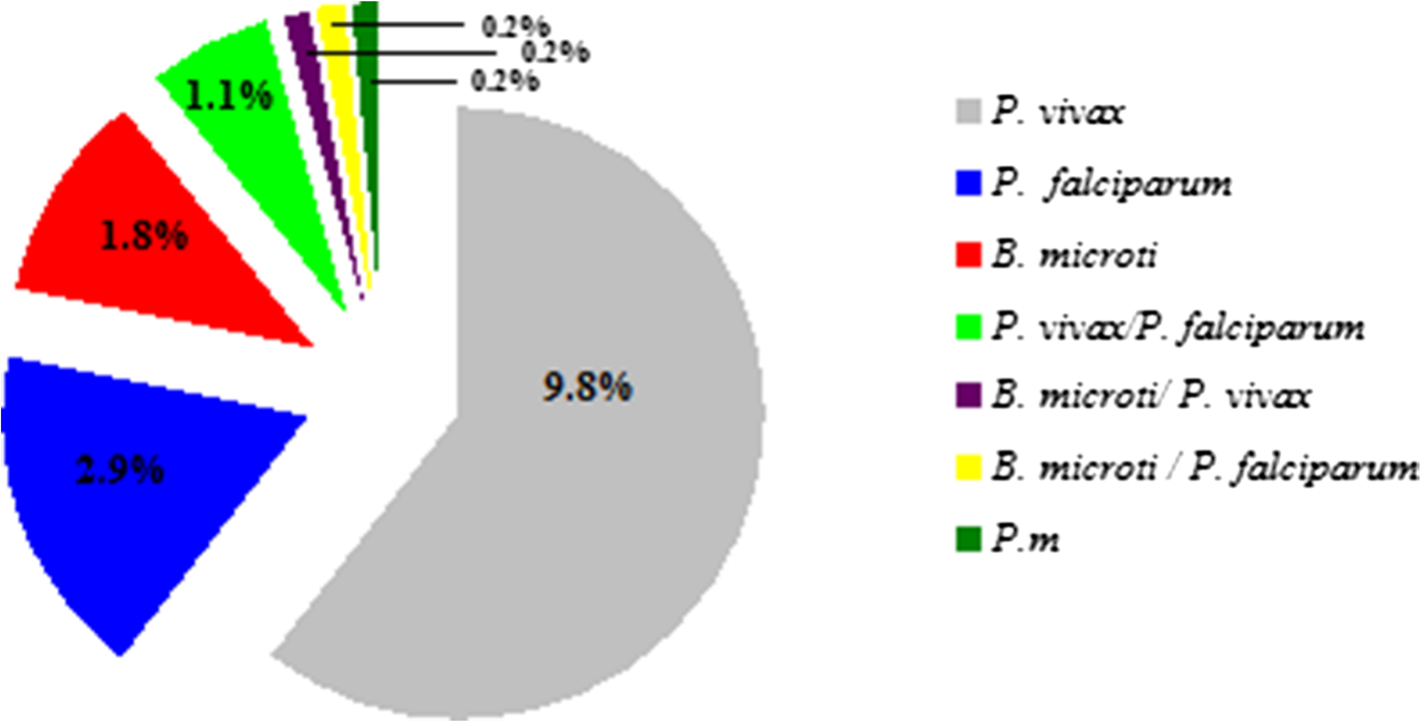
**Infection rate of *B. microti *** **and *Plasmodium *** **spp. in 449 suspected febrile patients in the area along the China-Myanmar border in the Yunnan province between April 2012 and June 2013.**

Four species of parasites taking residence in human erythrocyte (*B. microti, P. falciparum*, *P. vivax,* and *P. malariae*) were identified in 449 suspected febrile persons. Mono-infection with *B. microti, P. vivax*, *P. falciparum*, or *P. malariae* accounted for 1.8% (8/449), 9.8% (44/449), 2.9% (13/449), and 0.2% (1/449), respectively, of the total infections. The rate of mixed infections of *B. microti* and *P. falciparum* or *P. vivax* were both 0.2% (1/449), and mixed infections of *P. falciparum* and *P. vivax* accounted for 1.1% (5/449) (see Table 1 and Figure 1). The total prevalence of *B. microti* was 2.2% (10/449). One *P. falciparum* mono-infection case, five *P. vivax*, and eight *B. microti* cases were detected from microscopy screening negative samples. Two mixed infection cases of *B. microti* co-infection with *P. falciparum* or *P. vivax* were initially diagnosed as mono-infections of *Plasmodium* spp*.* by microscopy screening.

**Table 1:** ***B. microti *** **and *Plasmodium *** **spp. detection in 449 suspected febrile patients in the area along the China-Myanmar border in the Yunnan province between April 2012 and June 2013**

**Parasite**	**No. (%) persons**
*B. microti*	8 (1.8)
*B. microti / P. vivax*	1 (0.2)
*B. microti / P. falciparum*	1 (0.2)
*P. vivax*	44 (9.8)
*P. falciparum*	13 (2.9)
*P. vivax / P. falciparum*	5 (1.1)
*P. malariae*	1 (0.2)

### SSU rRNA sequence and phylogenetic analysis

**Figure 2:**
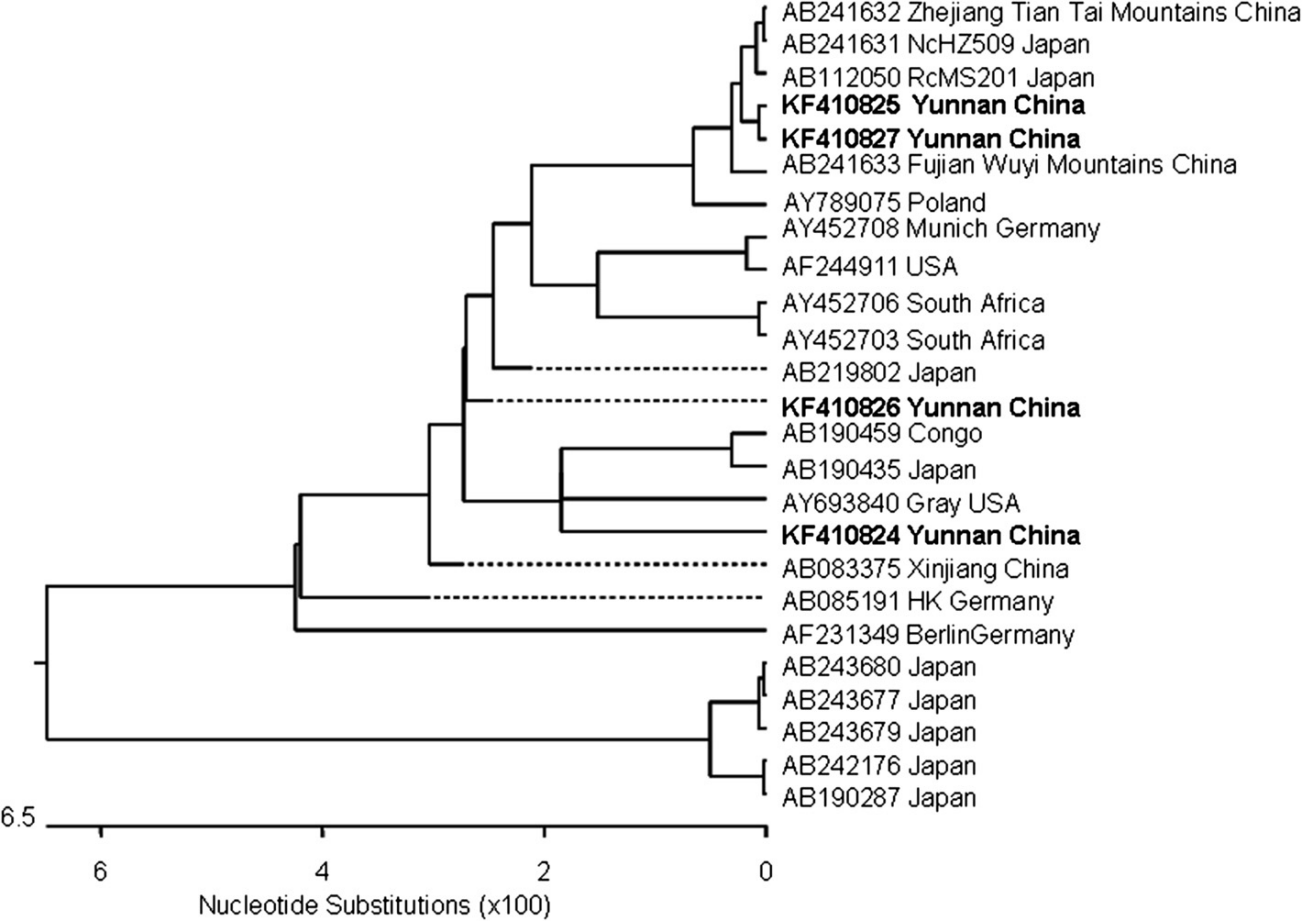
**Phylogenetic analysis of 18S small subunit SSU rRNA gene sequences of *B. microti *in the malaria-endemic area along the China-Myanmar border in the Yunnan province, April 2012 to June 2013.** Fragments of 18S SSU rRNA gene sequences of samples were analyzed by aligning with published homologous sequences of *B. microti* from different countries or regions*.* A phylogenetic tree was constructed on the basis of similarities by the MegAlign software (DNASTAR, Inc. Madison, WI, USA). Novel sequences identified in this study are indicated in **boldface**. Scale bar indicates nucleotide substitutions per site.

All the positive samples were sequenced. Sequence identity was confirmed by random basic local alignment search tool analysis of sequences in GenBank (http://blast.ncbi.nlm.nih.gov/). Novel sequences were deposited in GenBank with accession nos. KF410824-KF410827. Phylogenetic relationships of unique sequences amplified by using nested primers with corresponding reference sequences were constructed by using the neighbor-joining method using MegAlign software (DNASTAR, Inc. Madison, WI, USA). All sequences clustered with reference sequences of *B. microti* were from different countries or regions, which suggested that all sequences were species specific. Phylogenetic analysis showed that amplified products were species specific (see Figure 2).

### Morphological characteristics

**Figure 3:**
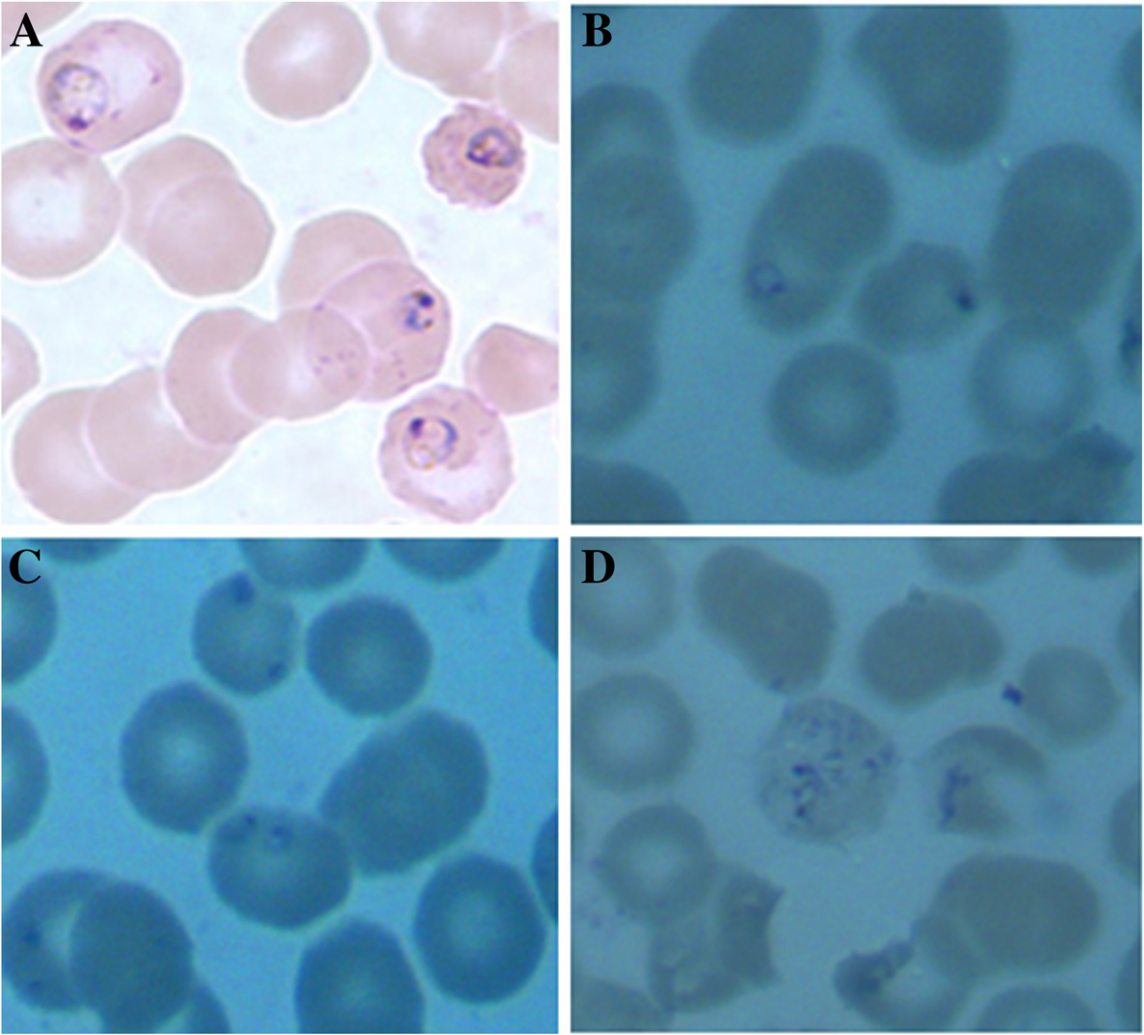
**Typical morphology of erythrocytic stages of *B. microti *in patients in the area along the China-Myanmar border in the Yunnan province, P.R. China.** Giemsa-stained thin blood films from field samples. **A**: Early trophozoite or ring form of *P. falciparum* and malaria pigment was scattered in the infected erythrocytes. **B**: Ring forms of *B. microti* in one erythrocyte without pigment. **C**: Two ring forms take residents in one erythrocyte **D**: Tetrads of merozoites that are arranged in a cross-like pattern (a so-called Maltese cross).

We observed the intro-erythrocytic cycle of the field samples in Giemsa-stained blood films from individuals that were identified by PCR as having *B. microti* infections. *Babesia* species resemble *P. falciparum*, however, *Babesia* spp. has several distinguishing features: the early trophozoite stage of *B. microti* is smaller than that of *P. falciparum* and the infected erythrocytes are without any pigments. And the late stage of trophozoites is pleomorphic and can be vacuolated. Ring forms of *B. microti* appeared smaller than that of *P. falciparum* and do not produce pigment (see Figure 3A), while early trophozoites of *P. falciparum* were larger and malaria pigment was scattered in the infected erythrocytes (see Figure 3B). Occasionally, two trophozoites or ring forms would take residence in one erythrocyte (see Figure 3C). Tetrads of merozoites that are arranged in a cross-like pattern (a so-called Maltese cross) being pathognomonic for babesiosis can be seen (see Figure 3D).

### Epidemiological data, clinical history

**Figure 4:**
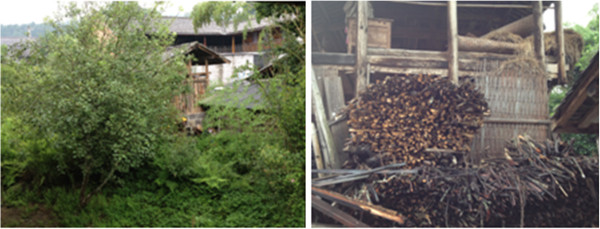
Area along the China-Myanmar border in the Yunnan province, P.R. China (which is hilly, and largely covered by primary and secondary rainforests) with babesiosis and malaria.

The demographic details of ten babesiosis patients detected by nested PCR from 449 individuals were recalled. All of these individuals were from the aforementioned study area (see Figure 4). The brief hospital records and data for clinical presentation, and whether outbound history was available, are shown in Table 2, but the treatment and outcome was not available. All of the patients were adults and complained of fever with chills and rigor before admission to hospital. We tracked two babesiosis cases as well and found that both of them recalled multiple tick bites in the recent past and also participated in outdoor activities in the mountains. One patient had even received blood transfusion and blood products for treatment of renal-malaria before because of infection of *P. falciparum.*

**Table 2:** Demographic and clinical data for patients of babesiosis at dates of admission, in the area along the China-Myanmar border in the Yunnan province, April 2012 to June 2013

**Demographic data (n = 10)**	
Age (years)	32.9 (8.2, 22–45)
Male gender	6 (60%)
Adults (>18 years)	10 (100%)
Clinical history and presentations (n = 10)	
Temperature at admission (°C)	37.8 (0.82, 36.0-38.8)
Outbound history	6 (60%)
Duration of illness before admission (days)	6.2 (9.4, 1–32)
Fever, chill, and rigor	10 (100%)

Data shown as n (% of total), or mean (SD; range) unless otherwise stated.

## Discussion

Between 1931 and 1944, Hung S.L. *et al.*[26] reported several human parasitemia in Chongqing, China, on protozoa taking residents in human erythrocytes, which was described as being similar to *P. falciparum*, but with fundamental differences of smaller ring forms and no pigments, and so it clearly indicated that it was *Babesia* spp. infection. These reports came 13 years earlier than the first case of human babesiosis was reported in a Yugoslavian farmer in 1957 [1], and strongly suggested that human *Babesia* spp. infection may have already existed widely in P.R. China. In fact, Yunnan, Inner Mongolia, Taiwan and Zhejiang, and other regions in P.R. China also have sporadic human babesiosis cases reported from 1984 to 2012 [5,8,27-30]. Sun Y *et al.*[31] reported that a *B. microti*-like rodent parasite was isolated from the tick, *I. persulcatus*, collected from the northern forest area of Heilongjiang province, P.R. China. Saito-Ito *et al.*[32] confirmed *Babesia* spp. infection of the Kobe strain presented in rodents in Zhejiang, Fujian, and Taiwan. But there are no reports of regional popularity on human babesiosis especilly in these natural foci areas in which *Babesia spp.* existed in the ticks or/and other reservoir hosts. Our study supports the hypothesis that human babesiosis caused by *B. microti* infection is emerging in areas along the China-Myanmar border in counties such as Tengchong in the Yunnan province, P.R. China, but can be ignored because of mix infection with *Plasmodium* spp. or low parasitemia.

Both *Plasmodium* and *Babesia* species are intraerythrocytic protozoans and they elicit similar inflammatory responses and clinical manifestations that differ markedly in severity. *Babesia* spp. co-infection with *P. falciparum* could not be ruled out in one case. The possibility of co-infection with babesial and malarial agents was well described in an infant from the Ivory Coast who was quite ill with parasitaemia of 35% [33]. Co-infection with both agents was reported in another rhesus monkey imported from Guangxi, P.R. China and the suspected infection of *B. microti*-like originated from primate-breeding facility [34]. Here, we detected two cases of febrile patients who were co-infected with *Plasmodium* spp. and *Babesia* spp. in malaria endemic area by molecular survey – it was unclear what the dominant parasite was and the microscopy detection of the parasite species of co-infections cases seem more difficult. It was reported that a rhesus monkey that was chronically infected with *B. microti* was able to control infection with *P. cynomolgi* better than naïve monkeys [35]. The suppression of *Plasmodium* spp. infection after chronic exposure to *B. microti* also warrants further investigation of a possible protective role of *Babesia* spp. infection on *Plasmodium spp*.

The new finding of mono-infection of *Babesia* and co-infection of *Babesia* spp. and *Plasmodium* spp. in the malaria-endemic area of the Tengchong county would bring new challenges to the prevention and controlling of these infectious diseases. It must also be noted that most antimalarial drugs, such as chloroquine, mefloquine, and artemisinin, have no effect on babesiosis [36]. Quinine and clindamycin, the former of which is often used for treatment of drug-resistant malaria, are the first-choice drugs against babesiosis [4,37]. Therefore, babesiosis in areas in which malaria is endemic might be misdiagnosed as drug-resistant malaria. From this standpoint, the identification of babesial parasite infection in the Yunnan province, where malaria is endemic, seems to deserve attention, considering that recent Asian cases of human babesiosis emerged in P. R. China and Japan, where *B. microti*-like parasites had earlier been identified in rodents. The importance of this finding in human co-infection of *Babesia* spp. and *Plasmodium* spp. is also reflected in case therapy as treatment of malaria patients depends on correct diagnosis of parasite species [38]. Ring forms of *Babesia spp.* may resemble those of *P. falciparum*[9]. Artemisinin-based combination therapies are the recommended first-line treatments of *falciparum* malaria in all countries where malaria transmitted. There are recent concerns that the efficacy of such therapies has declined on the Thailand-Cambodian and Thailand-Myanmar borders, historically sites of emerging antimalarial-drug resistance [39,40]. The morphology of *P. falciparum* and *B. microti* are similar especially in the ring form stage which is the main intraerythrocyte of both parasites so human Babesiosis may be misdiagnosed as *falciparum* malaria in these malaria endemic areas. Giving Artemisinin-based combination therapies to these babesiosis patients due to misdiagnosis would undoubtedly increase the pressure of artemisinin resistance in malaria-transmitted areas.

## Conclusions

Babesiosis has sometimes been diagnosed initially as malaria because of the similarity between the two diseases, which is the main reason why it is difficult to detect it in co-infections [41]. This report supports the hypothesis that babesiosis caused by *B. microti* is emerging along the China-Myanmar border in the Yunnan province, P.R. China, but it was ignored because of low parasitemia or mixed infection with *Plasmodium* spp. It’s crucial to apply more sensitive and specific diagnosis methods to set up rapid response mechanisms of emergency for the important infectious diseases in babesiosis and malaria syndemic areas, particularly in places which are weak in surveillance and response systems [42].

## Competing interests

The authors declare that they have no competing interests.

## Authors’ contributions

XZ conceived the study, collected the data and analyzed it, and drafted the manuscript. SGL and JZW revised the manuscript and provided intellectual input for the interpretation of the findings. SBC, BX, and HJZ conceived the project and provided technical support for data collection and analysis. JHC, HXZG and WH conceived the study and revised the manuscript. All authors read and approved the final manuscript.

## Supplementary Material

Additional file 1 Multilingual abstracts in the six official working languages of the United Nations. Click here for file

## References

[B1] Skrabalo Z Deanovic Z Piroplasmosis in man; report of a case Doc Med Geogr Trop 1957 9 11 16 13427667

[B2] Western KA Benson GD Gleason NN Healy GR Schultz MG Babesiosis in a Massachusetts resident N Engl J Med 1970 283 854 856 10.1056/NEJM197010152831607 4989787

[B3] Spielman A Clifford CM Piesman J Corwin MD Human babesiosis on Nantucket Island, USA: description of the vector, Ixodes (Ixodes) dammini, n. sp. (Acarina: Ixodidae) J Med Entomol 1979 15 218 234 439119 10.1093/jmedent/15.3.218

[B4] Vannier E Krause PJ Human babesiosis N Engl J Med 2012 366 2397 2407 10.1056/NEJMra1202018 22716978

[B5] Shih CM Liu LP Chung WC Ong SJ Wang CC Human babesiosis in Taiwan: asymptomatic infection with a Babesia microti-like organism in a Taiwanese woman J Clin Microbiol 1997 35 450 454 9003614 10.1128/jcm.35.2.450-454.1997 PMC229598

[B6] Shaio MF Lin PR A case study of cytokine profiles in acute human babesiosis Am J Trop Med Hyg 1998 58 335 337 9546414 10.4269/ajtmh.1998.58.335

[B7] Wei Q Tsuji M Zamoto A Kohsaki M Matsui T Shiota T Telford SR 3rd Ishihara C Human babesiosis in Japan: isolation of Babesia microti-like parasites from an asymptomatic transfusion donor and from a rodent from an area where babesiosis is endemic J Clin Microbiol 2001 39 2178 2183 10.1128/JCM.39.6.2178-2183.2001 11376054 PMC88108

[B8] Yao LN Ruan W Zeng CY Li ZH Zhang X Lei YL Lu QY Che HL Pathogen identification and clinical diagnosis for one case infected with Babesia Zhongguo Ji Sheng Chong Xue Yu Ji Sheng Chong Bing Za Zhi 2012 30 118 121 22908812

[B9] Hunfeld KP Hildebrandt A Gray JS Babesiosis: recent insights into an ancient disease Int J Parasitol 2008 38 1219 1237 10.1016/j.ijpara.2008.03.001 18440005

[B10] Gorenflot A Moubri K Precigout E Carcy B Schetters TP Human babesiosis Ann Trop Med Parasitol 1998 92 489 501 10.1080/00034989859465 9683900

[B11] Hildebrandt A Hunfeld KP Baier M Krumbholz A Sachse S Lorenzen T Kiehntopf M Fricke HJ Straube E First confirmed autochthonous case Of human Babesia microti infection in Europe Eur J Clin Microbiol Infect Dis 2007 26 595 601 10.1007/s10096-007-0333-1 17587072

[B12] Vannier E Gewurz BE Krause PJ Human babesiosis Infect Dis Clin North Am 2008 22 469 488 10.1016/j.idc.2008.03.010 18755385 PMC3998201

[B13] Kjemtrup AM Conrad PA Human babesiosis: an emerging tick-borne disease Int J Parasitol 2000 30 1323 1337 10.1016/S0020-7519(00)00137-5 11113258

[B14] Senanayake SN Paparini A Latimer M Andriolo K Dasilva AJ Wilson H Xayavong MV Collignon PJ Jeans P Irwin PJ First report of human babesiosis in Australia Med J Aust 2012 196 350 352 10.5694/mja11.11378 22432676

[B15] Mazigo HD Nuwaha F Wilson S Kinung'hi SM Morona D Waihenya R Heukelbach J Dunne DW Epidemiology and interactions of human immunodeficiency virus - 1 and schistosoma mansoni in sub-saharan africa Infect Dis Poverty 2013 2 2 10.1186/2049-9957-2-2 23849678 PMC3707091

[B16] Li XX Zhou XN Co-infection of tuberculosis and parasitic diseases in humans: a systematic review Parasit Vectors 2013 6 79 10.1186/1756-3305-6-79 23522098 PMC3614457

[B17] Tian LG Chen JX Wang TP Cheng GJ Steinmann P Wang FF Cai YC Yin XM Guo J Zhou L Zhou XN Co-infection of HIV and intestinal parasites in rural area of China Parasit Vectors 2012 5 36 10.1186/1756-3305-5-36 22330320 PMC3310850

[B18] Homer MJ Aguilar-Delfin I Telford SR 3rd Krause PJ Persing DH Babesiosis Clin Microbiol Rev 2000 13 451 469 10.1128/CMR.13.3.451-469.2000 10885987 PMC88943

[B19] Dantrakool A Somboon P Hashimoto T Saito-Ito A Identification of a new type of Babesia species in wild rats (Bandicota indica) in Chiang Mai Province, Thailand J Clin Microbiol 2004 42 850 854 10.1128/JCM.42.2.850-854.2004 14766871 PMC344436

[B20] Moore SJ Min X Hill N Jones C Zaixing Z Cameron MM Border malaria in China: knowledge and use of personal protection by minority populations and implications for malaria control: a questionnaire-based survey BMC Public Health 2008 8 344 10.1186/1471-2458-8-344 18828901 PMC2576233

[B21] Liu J Yang B Cheung WK Yang G Malaria transmission modelling: a network perspective Infect Dis Poverty 2012 1 11 10.1186/2049-9957-1-11 23849949 PMC3710080

[B22] Snounou G Viriyakosol S Zhu XP Jarra W Pinheiro L Do Rosario VE Thaithong S Brown KN High sensitivity of detection of human malaria parasites by the use of nested polymerase chain reaction Mol Biochem Parasitol 1993 61 315 320 10.1016/0166-6851(93)90077-B 8264734

[B23] Persing DH Mathiesen D Marshall WF Telford SR Spielman A Thomford JW Conrad PA Detection of Babesia microti by polymerase chain reaction J Clin Microbiol 1992 30 2097 2103 1500517 10.1128/jcm.30.8.2097-2103.1992 PMC265450

[B24] Medlin L Elwood HJ Stickel S Sogin ML The characterization of enzymatically amplified eukaryotic 16S-like rRNA-coding regions Gene 1988 71 491 499 10.1016/0378-1119(88)90066-2 3224833

[B25] Saito-Ito A Tsuji M Wei Q He S Matsui T Kohsaki M Arai S Kamiyama T Hioki K Ishihara C Transfusion-acquired, autochthonous human babesiosis in Japan: isolation of Babesia microti-like parasites with hu-RBC-SCID mice J Clin Microbiol 2000 38 4511 4516 11101588 10.1128/jcm.38.12.4511-4516.2000 PMC87629

[B26] SL H Notes on a species of malaria parasite finding from Bei-Bei National Medical Journal of China (Chongqing ed) 1944 19 571 573

[B27] Li JFMD Wang QF The discovery of human Babesiasis Chinese Journal of Veterinary Medicine 1984 6 19 20

[B28] Su GGn Zhao NF Ye YX A babesia case report Chinese Journal of Zoonose 2002 18 112 155

[B29] Shi ZBLZ Gao QR Ding YM One case of human babesia infection Chinese Journal o f Par asitology & Parasitic Diseases 1996 14 240

[B30] HX W One case of babesiosis in Kunming International Journal of Medical Parasitic Diseases 2012 39 190 192

[B31] Sun Y Liu G Yang L Xu R Cao W Babesia microti-like rodent parasites isolated from Ixodes persulcatus (Acari: Ixodidae) in Heilongjiang Province, China Vet Parasitol 2008 156 333 339 10.1016/j.vetpar.2008.05.026 18718720

[B32] Saito-Ito A Takada N Ishiguro F Fujita H Yano Y Ma XH Chen ER Detection of Kobe-type Babesia microti associated with Japanese human babesiosis in field rodents in central Taiwan and southeastern mainland China Parasitology 2008 135 691 699 18413002 10.1017/S0031182008004356

[B33] Vermeil C Menut J Miegeville M Cruziat J Julienne F Morin O Roger AP Marjolet M Bouillard C Babesiasis, pediatric malaria: does confusion exist in Africa? Bull Soc Pathol Exot Filiales 1983 76 797 804 6368023

[B34] Voorberg-vd Wel A Kocken CH Zeeman AM Thomas AW Detection of new Babesia microti-like parasites in a rhesus monkey (Macaca mulatta) with a suppressed Plasmodium cynomolgi infection Am J Trop Med Hyg 2008 78 643 645 18385363

[B35] van Duivenvoorde LM van der Voorberg Wel A Van der Werff NM Braskamp G Remarque EJ Kondova I Kocken CH Thomas AW Suppression of Plasmodium cynomolgi in rhesus macaques by coinfection with Babesia microti Infect Immun 2010 78 1032 1039 10.1128/IAI.00921-09 20048045 PMC2825946

[B36] Marley SE Eberhard ML Steurer FJ Ellis WL McGreevy PB Ruebush TK 2nd Evaluation of selected antiprotozoal drugs in the Babesia microti-hamster model Antimicrob Agents Chemother 1997 41 91 94 8980761 10.1128/aac.41.1.91 PMC163666

[B37] Centers for Disease Control (CDC), USA, Weekly report Clindamycin and quinine treatment for Babesia microti infections MMWR Morb Mortal Wkly Rep 1983 32 65 66 72 6405180

[B38] Lalloo DG Shingadia D Pasvol G Chiodini PL Whitty CJ Beeching NJ Hill DR Warrell DA Bannister BA UK malaria treatment guidelines J Infect 2007 54 111 121 10.1016/j.jinf.2006.12.003 17215045

[B39] Htut ZW Artemisinin resistance in Plasmodium falciparum malaria N Engl J Med 2009 361 1807 1808 author reply 1808 19877309

[B40] Phyo AP Nkhoma S Stepniewska K Ashley EA Nair S McGready R Ler Moo C Al-Saai S Dondorp AM Lwin KM Emergence of artemisinin-resistant malaria on the western border of Thailand: a longitudinal study Lancet 2012 179 1960 1966 22484134 10.1016/S0140-6736(12)60484-X PMC3525980

[B41] Ansa GA Walley JD Siddiqi K Wei X Assessing the impact of TB/HIV services integration on TB treatment outcomes and their relevance in TB/HIV monitoring in Ghana Infect Dis Poverty 2012 1 13 10.1186/2049-9957-1-13 23849044 PMC3710204

[B42] Zhou XN Bergquist R Tanner M Elimination of tropical disease through surveillance and response Infect Dis Poverty 2013 2 1 10.1186/2049-9957-2-1 23849433 PMC3707090

