# Supplementary material for: Co-infections with Babesia microti and Plasmodium parasites along the China-Myanmar border
Source: Infect Dis Poverty. 2013 Oct 3;2:24. doi: 10.1186/2049-9957-2-24 (PMC3819642; doi:10.1186/2049-9957-2-24)
Supplement: Additional file 1 — Multilingual abstracts in the six official working languages of the United Nations. [file 2049-9957-2-24-S1.pdf]

Translation of the abstract into the six official working languages of the United Nations

الالتهابات بالبابيسيا ميكروتي وطفيليات البلازموديوم على طول الحدود بين الصين وميانمار

زيا تزو، شنغ-جووّ لى، شين-بو تشن، جيا-زى وانج، بن زو، وهيو-جن تزو، وهونج –تزيانج تزو جي، وهيو ويي، وهيو-جن تشن

الملخص

خلفية: يُعد البابيوزيس خطراً ضمن المخاطر الصحية الناشئة في العديد من أجزاء العالم. ومع ذلك، لا يُعرف إلا القليل عن انتشار البابيزية في البلدان التي تستوطن فيها الملاريا. إن المنطقة الواقعة على طول الحدود بين الصين وميانمار فى يونان هي المنطقة المستوطنة الرئيسية لمرض الملاريا في جمهورية الصين الشعبية، ومع ذلك، فإن حالات العدوى البشرية الناجمة عن البابيزية الميكروتية العكبرية (*B. microti*) غير مُتعارف عليها في هذه المنطقة، وليس لها موقع بعد على خريطة الإصابة بالأمراض المعدية المشتركة.

الطرق والأساليب: تم إجراء الدراسة في المنطقة التي تستوطن فيها الملاريا على طول الحدود بين الصين وميانمار في يونان، وذلك في الفترة ما بين أبريل 2012 ويونيو 2013 لفهم أوضاع وحالات الإصابة بالعدوى عن طريق (*B. microti*)، وقد تم تحديد أربعة أنواع من الطفيليات، بما في ذلك البابيزية العكبرية (*B. microti*)، والمتصورة المنجلية (*P. falciparum*)، والمتصورة النشيطة (*P. vivax*)، والمتصورة الملاريية (*P. malariae*)، وأخذت عينات من 449 شخص مُشتبه بهم يُعانون من الحُمي. وقد تم الكشف عنها بواسطة متداخلة تفاعل البلمرة (PCR) مُقارنة بوحدة صغيرة من وحدات سلسلة الحمض النووي الريبي (RNA) المكون من (*B. microti*) البابيزية العكبرية والمتصورة المنجلية.

النتائج: من بين كل العينات التي تم جمعها، حصلنا على النتائج التالية من المرضى الذين يُعانون من الحُمى الناتجة عن الإصابة بـ (*B. microti*) العكبرية، والمتصورة النشيطة (*P. vivax*)، والمتصورة المنجلية (*P. falciparum*)، والمتصورة الملاريية (*P. malariae*) التي تُمثل 1.8٪ (8/449)، و9.8٪ (44/449)، و2.9 ٪ (13/449)، و 0.2٪ (449/1) على التوالي. وكان معدل الإصابات المُختلطة من (*B. microti*) العكبرية مع المتصورة المنجلية أو المتصورة النشيطة تُشكل 0.2٪ (1/449)، والإلتهابات المختلطة من المتصورة المنجلية والمتصورة النشيطة تُشكل 1.1٪ (5/449).

الاستنتاجات: يدعم هذا التقرير فرضية أن البابيزية التي تُسببها (*B. microti*) العكبرية تنتشر على طول الحدود بين الصين وميانمار في إقليم يونان، جمهورية الصين الشعبية، ولكن يُمكن تجاهلها بسبب انخفاض الطفيليات أو العدوى المختلطة مع المتصورة البلازموديومية. وهناك حاجة إلى استحداث طرق التشخيص أكثر حساسية وطرق تحديد تشخيصية لإيجاد آلية سريعة الاستجابة في حالات الطوارئ لكل من البابيزية والملاريا في مناطق الانتشار التابعة لإطار التعاون.

Translated from English version into Arabic by Freelanceabunar, through

TRANSLATORS
WITHOUT BORDERS

田鼠巴贝虫与疟原虫在云南中-缅边境地区共感染研究

周霞，李胜国，陈绅波，王加志，徐斌，周何军，诸葛洪祥，陈军虎，胡薇

摘要

引言：巴贝虫病是一种在世界各地均有分布的威胁人类健康的新发传染病。然而，目前鲜有对疟疾流行地区巴贝虫病患病率的研究。云南省中-缅边境地区是我国主要疟疾流行区，巴贝虫病在该地区的感染情况不详，其与疟疾的共感染现象亦不清楚。

方法：为了解田鼠巴贝虫病感染情况，我们对2012年四月至2013年六月期间云南中-缅边境沿线疟疾流行区的449名发热病人用针对田鼠巴贝虫和疟原虫核糖体小亚基核糖核酸（RNA）的巢式PCR技术进行了分子生物学检测分析。

结果：在所有采集的发热病人血样中，单个感染为田鼠巴贝虫、间日疟原虫、恶性疟原虫和三日疟原虫的比例分别为1.8% (8/449)、9.8% (44/449)、2.9% (13/449)和0.2% (1/449)。田鼠巴贝虫与恶性疟原虫、田鼠巴贝虫与间日疟原虫混合感染比例均为0.2% (1/449)，间日疟原虫与恶性疟原虫混合感染比例为 1.1% (5/449)。

结论：该研究表明由感染田鼠巴贝虫导致的巴贝虫病已出现在我国主要疟疾流行区，云南省中-缅边境地区，但由于巴贝虫病的低原虫血症和巴贝虫与疟原虫的混合感染而被忽视。为应对巴贝虫病和疟疾共同流行区防控工作，急需研究更加敏感特异的巴贝虫病诊断方法。

Translated from English version into Chinese by Zhou Xia

Les co-infections parasitaires par *Babesia microti* et *Plasmodium* le long de la frontière Chine-Myanmar

Xia Zhou, Sheng-Guo Li, Shen-Bo Chen, Jia-Zhi Wang, Bin Xu, He-Jun Zhou, Hong-Xiang Zhu Ge, Hu Wei, Jun-Hu Chen

Résumé

Rappel des faits : La babésiose est un risque de santé émergent dans plusieurs régions du monde. Cependant, on connaît peu de chose concernant la prévalence du *Babesia* en pays d'endémie de paludisme. La zone le long de la frontière entre la Chine et de Myanmar dans le Yunnan est une zone principale d'endémie de paludisme en République populaire de Chine. Cependant, l'infection humaine par le *Babesia microti* (*B. microti*) n'est pas reconnue dans cette région et son profil de co-infection avec le Plasmodium n'est pas encore clair.

Méthodes : Pour comprendre son profil de co-infections avec le B. microti, nous avons entrepris une enquête dans la région de paludisme endémique située le long de la frontière entre la Chine et le Myanmar au Yunnan entre avril 2012 et juin 2013. Quatre espèces de parasites comprenant les *B. microti, Plasmodium falciparum* (*P. falciparum*), *P. vivax,* and *P. malariae,* ont été identifiées parmi les 449 personnes fébriles suspectes et ont été détectées par amplification en chaîne par polymérase (ACP) sur la base de la présence d'une petite sous-unité ribosomique d'acide ribonucléique (ARN) de gènes de *B. microti* et de *Plasmodium*.

Résultats : Sur l'ensemble des échantillons de patients fébriles, une mono-Infection à *B. microti, P. vivax*, *P. falciparum*et *P. malariae* a été retrouvée respectivement dans 1,8 % (8/449), 9,8 % (44/449), 2,9 % (13/449) et 0,2 % (1/449) des échantillons. Le taux des infections mixtes dut à *B. microti* et *P. falciparum* ou *P. vivax* représentait 0,2 % (1/449) chacun et celui des infections mixtes à P.*falciparum* et *P. vivax* 1,1 % (5/449).

Conclusions : Ce rapport confirme l'hypothèse que la babésiose causée par *B. microti* fait son apparition le long de la frontière entre la Chine et le Myanmar dans la province du Yunnan en République populaire de Chine, mais peut être ignorée en raison de la faible parasitémie ou infection mixte à *B. microti et Plasmodium*. Des méthodes de diagnostic plus sensibles et spécifiques sont nécessaires pour avoir un moyen de réponse rapide en cas d'urgence pour les zones de co-prévalence de babésiose et de malaria.

Translated from English version into French by Michele Lemaire, through

TRANSLATORS
WITHOUT BORDERS

Ко-инфекции паразитами *Babesia microti* и *Plasmodium* в регионах вдоль границы Китая и Мьянмы

Xia Zhou, Sheng-Guo Li, Shen-Bo Chen, Jia-Zhi Wang, Bin Xu, He-Jun Zhou, Hong-Xiang Zhu Ge, Hu Wei , Jun-Hu Chen

Резюме

Общие сведения: Бабезиозы представляют растущую угрозу здоровью населения нескольких регионах мира. Однако немногое известно о распространенности видов *Babesia* в эндемичных по малярии странах. Регион вдоль границы Китая и Мьянмы в провинции Юннань представляет собой основную эндемичную по малярии зону в Китайской Народной Республике, однако инфицирование населения *Babesia microti* (*B. microti*) в этом регионе не считается имеющим важное значение, а его профиль в качестве ко-инфекции до настоящего времени остается неясным.

Методы: С целью изучения профиля ко-инфицирования *B. microti* мы предприняли исследования в эндемичной по малярии зоне вдоль границы Китая и Мьянмы в провинции Юннань в период с апреля 2012 г. по июнь 2013 г. Четыре вида паразитов, включая *B. microti, Plasmodium falciparum* (*P. falciparum*), *P. vivax,* и *P. malariae,* были идентифицированы у 449 пациентов с лихорадкой методом полимеразной цепной реакции (ПЦР) с использованием РНК рибосом *B. microti* и *Plasmodium*.

Результаты: Среди всех проб, полученных от пациентов с лихорадкой, моно-инфекции *B. microti, P. vivax*, *P. falciparum* и *P. malariae* составили 1,8% (8/449), 9,8% (44/449), 2,9% (13/449) и 0,2% (1/449), соответственно. Смешанные инфекции *B. microti* и *P. falciparum* или *P. vivax* составили 0,2% (1/449), а смешанные инфекции *P. falciparum* и *P. vivax* – 1,1% (5/449).

Выводы: Данные этого отчета свидетельствуют в поддержку гипотезы о том, что бабезиоз, возбуждаемый *B. microti* , хоть и распространен в провинции Юннань вдоль границы с Мьянмой, можно игнорировать по причине низкой паразитемии или наличия инфекций совместных с *Plasmodium*. Необходимы более чувствительные и специфичные методы диагностики для принятия быстрых и действенных мер против бабезиоза в регионах, эндемичных по малярии.

Translated from English version into Russian by Natalie, through

TRANSLATORS
WITHOUT BORDERS

Co-infecciones entre parásitos de *Babesia microti* y *Plasmodium* a lo largo de la frontera entre China y Myanmar

Xia Zhou, Sheng-Guo Li, Shen-Bo Chen, Jia-Zhi Wang, Bin Xu, He-Jun Zhou, Hong-Xiang Zhu Ge, Hu Wei , Jun-Hu Chen

Resumen

Antecedentes: La babesiosis es un nuevo riesgo para la salud en varias partes del mundo. Sin embargo, se sabe poco sobre la tasa de prevalencia de *Babesia* en países donde la malaria es endémica. La región de Yunnan a lo largo de la frontera entre China y Myanmar es una importante zona endémica de malaria en la República Popular China. Sin embargo, no se ha reconocido el contagio humano con el *Babesia microti* (*B. microti*) en esta región y su perfil de co-infección no está claro aún.

Métodos: para conocer su perfil de co-infecciones con *B. microti*, realizamos nuestra investigación en Yunnan, la zona endémica de malaria a lo largo de la frontera entre China y Myanmar, entre abril de 2012 y junio de 2013. Se identificaron cuatro especies de parásitos, incluyendo *B. microti, Plasmodium falciparum* (*P. falciparum*), *P. vivax,* y *P. malariae,* entre 449 personas con sospecha de estado febril cuyos casos fueron detectados mediante la técnica de reacción en cadena de la polimerasa (PCR) anidada a base de genes de pequeñas subunidades de ácido ribonucléico ribosomal (RNA) de *B. microti* y *Plasmodium*.

Resultados: De todas las muestras recogidas entre los pacientes en estado febril, la mono-infección con *B. microti, P. vivax*, *P. falciparum*, y *P. malariae* supuso el 1,8% (8/449), el 9,8% (44/449), el 2,9% (13/449), y el 0,2% (1/449) de los casos, respectivamente. El índice de infecciones mixtas de *B. microti* con *P. falciparum* o *P. vivax* son de 0,2% los dos (1/449), y las infecciones mixtas de *P. falciparum* y *P. vivax* supusieron el 1,1% (5/449).

Conclusiones: este estudio apoyo la hipótesis de que la babesiosis causada por *B. microti* está apareciendo a lo largo de la frontera entre China y Myanmar en la provincia de Yunnan, en la República Popular China, pero puede ignorarse debido a la baja parasitemia o infección mixta con *Plasmodium*. Se necesitan métodos de diagnosis más precisos y específicos para encontrar el mecanismo de respuesta rápida en los casos de aparición en las áreas de co-prevalencia de babesiosis y malaria.

Translated from English version into Spanish by Bagueda, through

TRANSLATORS
WITHOUT BORDERS
